# PACT: a pipeline for analysis of circulating tumor DNA

**DOI:** 10.1093/bioinformatics/btad489

**Published:** 2023-08-07

**Authors:** Jace Webster, Ha X Dang, Pradeep S Chauhan, Wenjia Feng, Alex Shiang, Peter K Harris, Russell K Pachynski, Aadel A Chaudhuri, Christopher A Maher

**Affiliations:** McDonnell Genome Institute, Washington University in St. Louis, MO 63108, United States; McDonnell Genome Institute, Washington University in St. Louis, MO 63108, United States; Siteman Cancer Center, Washington University in St. Louis, MO 63110, United States; Department of Medicine, Washington University School of Medicine, St. Louis, MO 63110, United States; Department of Radiation Oncology, Washington University School of Medicine, St. Louis, MO 63110, United States; Department of Radiation Oncology, Washington University School of Medicine, St. Louis, MO 63110, United States; Department of Radiation Oncology, Washington University School of Medicine, St. Louis, MO 63110, United States; Siteman Cancer Center, Washington University in St. Louis, MO 63110, United States; Department of Medicine, Washington University School of Medicine, St. Louis, MO 63110, United States; Siteman Cancer Center, Washington University in St. Louis, MO 63110, United States; Department of Radiation Oncology, Washington University School of Medicine, St. Louis, MO 63110, United States; Department of Genetics, Washington University School of Medicine, St. Louis, MO 63110, United States; Department of Biomedical Engineering, Washington University in St. Louis, MO 63130, United States; Department of Computer Science and Engineering, Washington University in St. Louis, MO 63130, United States; McDonnell Genome Institute, Washington University in St. Louis, MO 63108, United States; Siteman Cancer Center, Washington University in St. Louis, MO 63110, United States; Department of Medicine, Washington University School of Medicine, St. Louis, MO 63110, United States; Department of Biomedical Engineering, Washington University in St. Louis, MO 63130, United States

## Abstract

**Motivation:**

Detection of genomic alterations in circulating tumor DNA (ctDNA) is currently used for active clinical monitoring of cancer progression and treatment response. While methods for analysis of small mutations are more developed, strategies for detecting structural variants (SVs) in ctDNA are limited. Additionally, reproducibly calling small-scale mutations, copy number alterations, and SVs in ctDNA is challenging due to the lack to unified tools for these different classes of variants.

**Results:**

We developed a unified pipeline for the analysis of ctDNA [Pipeline for the Analysis of ctDNA (PACT)] that accurately detects SVs and consistently outperformed similar tools when applied to simulated, cell line, and clinical data. We provide PACT in the form of a Common Workflow Language pipeline which can be run by popular workflow management systems in high-performance computing environments.

**Availability and implementation:**

PACT is freely available at https://github.com/ChrisMaherLab/PACT.

## 1 Introduction

Identification of genomic variants in circulating tumor DNA (ctDNA) has emerged as a promising method for non-invasive monitoring of cancer progression and treatment response. This non-invasive monitoring is particularly beneficial in metastatic disease as ctDNA originating from both primary and secondary tumor sites can be found within a single blood sample. Despite low ctDNA abundance and expected allele frequencies, deep targeted sequencing has been successfully used to improve sensitivity (Corcoran and Chabner 2018) for detecting single-nucleotide variants (SNVs). Structural variants (SVs) are a major class of genomic drivers of cancer progression (Dang *et al.* 2020a) but their use in non-invasive applications remains limited due to the challenges of accurately detecting the wide variety of possible complex genomic rearrangements.

When attempting to overcome the limitations of current ctDNA SV callers, while also identifying copy number alterations (CNAs) and small mutations, users often resort to *ad hoc* or proprietary approaches. This leads to time-consuming analyses and inhibits reproducibility in the field, in part because of the variety of possible customizable tools and parameters required. A few tools have been developed for SV detection in ctDNA; however, each of the identified tools has crucial limitations inhibiting their ability to identify clinically relevant events (Supplementary Table S1). For example, none of them accept matched germline control data, which is critical for differentiating artifacts, germline, and somatic events.

To address these limitations and promote the accurate and reproducible detection of SVs, CNAs, and small mutations in ctDNA, we developed an open-source unified Pipeline for the Analysis of ctDNA (PACT).

## 2 Methods

PACT is a standardized ctDNA pipeline for detection of SVs, CNAs, and small mutations. It is designed for reproducibility in high-performance computing environments capable of processing large numbers of samples and can be run by popular workflow management systems.

**Figure 1.**
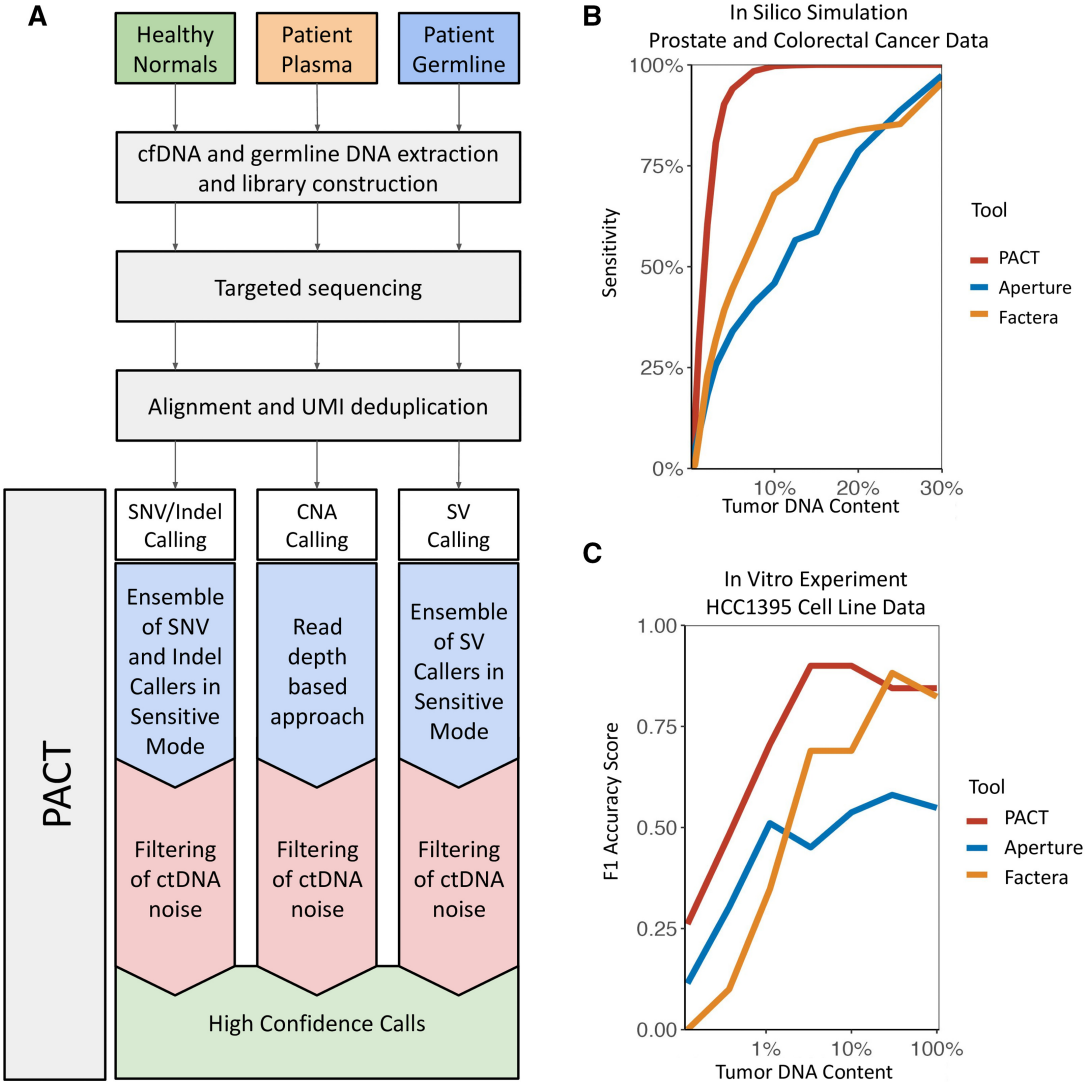
Summary of PACT workflow and benchmarking. (A) Overview of PACT workflow. (B) *In silico* and (C) *in vitro* benchmarking summaries.

PACT consists of methods for detection of small mutations, CNAs, and SVs independently (Fig. 1A and Supplementary Fig. S1; expanded in Supplementary Methods). Briefly, each variant calling strategy begins with the creation of an initial list of candidates nominated using an ensemble of tools for variant calling. Where possible, each tool is run using relaxed filtering criteria to increase sensitivity (Supplementary Methods). To obtain high specificity, normalization and/or filtering strategies are applied to all nominated variants based on expected noise in ctDNA caused in part by deep sequencing and low allele frequencies (often <1%) (Corcoran and Chabner 2018). All workflows accept sequencing data from matched controls and from a panel of unmatched, healthy individuals to aid in removing non-somatic events.

For SVs, consensus calls are first genotyped against both matched and unmatched controls to remove artifactual calls and germline events. After comparisons to controls are made, regional filters require at least one breakpoint of an SV to originate in a region targeted by the sequencing panel to ensure relevance and correct mapping, while removing calls from genomic regions with known high false positive rates. Finally, multiple forms of evidence (split-read and discordant paired-end read) are required for final SV nomination.

## 3 Results

Although SNV and CNA strategies for ctDNA are usually considered robust, SV detection has traditionally been challenging. While PACT integrates callers of various variant types, its major focus is on improving SV analysis in ctDNA. Here, we benchmark our tool using (i) patient data, (ii) an *in silico* simulation, and (iii) a dilution experiment, while comparing against other ctDNA SV callers including SViCT, Factera, and Aperture (Newman *et al.* 2014, Gawroński *et al.* 2019, Liu *et al.* 2021).

First, we applied each tool to ctDNA samples from a published cohort of 40 prostate cancer patients and found that only PACT and Aperture detected all published SVs (Supplementary Fig. S2) (Dang *et al.* 2020a). Precision was not assessed due to the lack of gold standard positive controls. Similarly, only PACT and Aperture identified all expected SVs in a public cfDNA reference dataset (SRA: SRR8551545). However, Aperture reported 1636 unvalidated SVs in the reference data (1623 more than the next highest tool, Factera), suggesting poor precision (Supplementary Fig. S3).

Second, we performed an *in silico* simulation with tumor data from four prostate (Dang *et al.* 2020a) and five colorectal (Dang *et al.* 2020b) cancer patients (Supplementary Table S2). Sequencing reads from tumor and respective matched controls were combined to simulate ctDNA content ranging from 0.1% to 30%, bounded by the tumor purity of original samples. At each dilution, PACT achieved the highest sensitivity (Fig. 1B). Specificity was not assessed as validation of novel calls could not be performed; however, we observed that Aperture and Factera consistently had the most candidates (i.e. approximately 13× and 8× more than PACT at 7.5% tumor DNA content; Supplementary Fig. S4), suggesting potentially poor precision.

Our third evaluation was performed using an *in vitro* dilution experiment of the well characterized breast cancer cell line (HCC1395) and its matched control cell line (HCC1395BL). We mixed these cells to create diluted samples with 0.1%–100% tumor content. Targeted sequencing of 26 validated SVs was then performed. We found that PACT achieved the highest sensitivity and F1 accuracy scores and was the only tool to achieve >90% sensitivity at all dilutions >3% (Fig. 1C and Supplementary Fig. S5). At the lowest detectable level (0.12% tumor content), PACT, Aperture, and Factera achieved 15%, 10%, and 0% sensitivity, respectively.

Together, these results suggest that PACT is both more sensitive and more precise than other ctDNA SV callers. By including SNV and CNA workflows within PACT and distributing the pipeline in a standardized workflow language, PACT is well suited for improving accuracy and reproducibility in ctDNA analysis, with potential clinical applications.

## Supplementary Material

btad489_Supplementary_Data
