# Supplementary material for: PACT: a pipeline for analysis of circulating tumor DNA
Source: Bioinformatics. 2023 Aug 7;39(8):btad489. doi: 10.1093/bioinformatics/btad489 (PMC10415172; doi:10.1093/bioinformatics/btad489)
Supplement: btad489_Supplementary_Data [file btad489_supplementary_data.docx]

**Supplementary Materials for**

**PACT: A pipeline for analysis of circulating tumor DNA**

Jace Webster, Ha X. Dang, Pradeep S. Chauhan, Wenjia Feng, Alex Shiang, Peter K. Harris, Russell K. Pachynski, Aadel A. Chaudhuri, Christopher A. Maher

**Supplementary Methods**

**PACT Workflows**

***SV workflow***

The PACT SV workflow can broadly be divided into two steps: 1) Creation of a broad list of SV candidates using relaxed filtering criteria and 2) filtering of candidates to reduce ctDNA-related noise. The first step in PACT begins by making initial somatic structural variant (SV) calls using Delly, Lumpy, and Manta in sensitive mode (Rausch *et al.*, 2012; Layer *et al.*, 2014; Chen *et al.*, 2016). All three callers are commonly used in studies of SVs, although none of them were specifically designed to be used on cell-free DNA (cfDNA). However, we adapted these tools for cfDNA by using relaxed parameters that allow sensitive reporting of SVs with low levels of read support. Delly calls are generated using default settings and the `*delly call*` command, however this command is not followed by the `*delly filter`* command normally recommended in the tool’s documentation, so that low-frequency SVs are not inadvertently removed at this stage. Lumpy calls are made using the `*lumpyexpress`* command, with the default minimum weight lowered to 3 by using the *`-m*` parameter. Manta is run using default settings, however only the candidateSV.vcf output file is used for downstream analysis, rather than relying on Manta’s built-in filtering that gets applied to the final somaticSV.vcf and diploidSV.vcf output files.

Consensus initial SV calls are then identified by merging initial SV candidates using SURVIVOR (Jeffares *et al.*, 2017). In our analyses we modified SURVIVOR’s default settings based on our experience applying SURIVOR to clinically relevant SVs (*max-distance-to-merge=100*, *minimum-sv-size=200*, *same-strand=false*, and *estimate-sv-distance=false*, all of which can be further modified by the user). Initial consensus SVs represent highly sensitive collection of SVs supported by multiple callers. The vcf file containing consensus calls is then modified with a custom script to ensure compatibility with downstream tools.

To achieve a high level of specificity, PACT preforms various filters to remove initial consensus SV calls that are likely false positives. First, targeted region-based filtering is performed to retain SV calls that originate from regions targeted by the targeted panel. A +/-200bp wingspan is automatically added to targeted regions to ensure that SVs with breakpoints that are located immediately adjacent to targeted regions are also retained.

Additional region-based filtering is performed to remove consensus SV calls that originate from genomic regions that are difficult to align to and tend to have high false positive rates. This is done by 1) immediately removing any consensus SV with a breakpoint that fails in a “blacklisted” region and 2) removing consensus SVs that has >1 breakpoint that falls in a low-complexity genomic region. In our benchmarking, blacklisted regions were based on the blacklist bed file provided by 10x Genomics at <http://cf.10xgenomics.com/supp/genome/hg19/sv_blacklist.bed> and low-complexity regions were taken from <https://github.com/lh3/varcmp/raw/maser/scripts/LCR-hs37d5.bed.gz> . We found that these inputs worked well in our benchmarking, but users can supply alternative regions using the “*neither_region” and “notboth_region”* parameters.

Next, PACT performs normal filtering to eliminate SV calls with evidence suggesting they are likely germline event or systematic (sequencing/alignment) artifacts. To do this, consensus SV calls from individual cfDNA samples across a patient cohort are merged and re-genotyped across matched controls and cfDNA samples from healthy individuals. *Svtools sort* command was used for sorting and *svtools lmerge* command was used for merging SV calls across samples (input as VCF format) to generate cohort-wide SV calls (also in VCF format)(Larson *et al.*, 2019). Cohort-wide SV calls were then subsequently genotyped across all samples and matched controls using SVTyper (Chiang *et al.*, 2015). Additionally, the user-supplied panel of healthy normals (sequenced with the same targeted panel) is genotyped using the cohort-wide VCF. The panel of healthy normal is expected to contain cfDNA sequencing data from healthy individuals, if available. In the case where a true panel of healthy normals are not available, the healthy normal panel may be substituted with a panel comprised of all available matched control samples (though they are not truly “healthy normal” samples). In this case, we recommend creating a panel of “normals” composed of any available matched controls, instead of using healthy individuals. Candidate SVs are then filtered based on genotyping results to remove those with supporting reads found in the panel of normal or in each sample’s respective matched control. Additionally, PACT retains only consensus SV calls with multiple types of read support in cfDNA samples (at least 1 supporting split-read and 1 supporting discordant paired-end read), requiring at least >2 total supporting reads. If either breakpoint from an SV overlaps with an optionally supplied “whitelist” region bed file, the requirement for two forms of evidence (split-read and discordant paired-end read) is waived, but the minimum read support threshold must still be met.

Finally, to help users interpret the SVs, PACT performs SV annotation using *snpEff* (Cingolani *et al.*, 2012). Final output also includes additional useful information, including which of the SV callers (Delly, Lumpy and Manta) originally reported the SV, whether the event corresponds to the optionally provided whitelist, and the number of supporting split-read and discordant read-pairs that were found. The final result of the SV workflow is a highly confident list of annotated SV calls presented in a standard bedpe format, designed to be compatible with downstream analysis tools for easy interpretation.

***SNV/Indel workflow***

SNV calling begins by generating candidate somatic calls using Mutect, Strelka, VarScan and Pindel (Cibulskis *et al.*, 2013; Kim *et al.*, 2018; Koboldt *et al.*, 2009; Ye *et al.*, 2009). Each variant caller accepts a variety of unique parameters. Where possible, the PACT workflow allows full customization of input values for parameters that are passed to each tool, but attempts to provide reasonable defaults (documented at <https://github.com/ChrisMaherLab/PACT>) based on observed metrics in ctDNA where possible. Additionally, PACT accepts a list of whitelisted variants in VCF format which are genotyped using GATK’s *HaplotypeCaller* (Poplin *et al.*, 2017). In our testing, we used the whitelist VCF that can be downloaded from the DoCM database (Ainscough *et al.*, 2016). All candidate calls are then combined and then decomposed using Vt’s *decompose* function (Tan *et al.*, 2015).

The decomposed VCF is next annotated using *vep* (McLaren *et al.*, 2016) and read counts are standardized by re-calculating the read depth for each SNV in both the tumor and matched control sample using the *bam-readcount* tool (Khanna *et al.*, 2022). This allows for a standardized read depth measurement, rather than relying on reported read counts determined by individual callers. Filtering is then performed and is based on SNV frequency in gnomAD (https://gnomad.broadinstitute.org), mapping quality, read depth, and allele frequency. All thresholds include default values based on ctDNA quality control metrics observed by our group and can be found on the project’s GitHub page (https://github.com/ChrisMaherLab/PACT). Finally, to address the large number of likely false positives that occur as a result of high sequencing depths combined with low expected variant allele frequencies, background error suppression is performed by genotyping candidate SNVs/indels in the user-provided panel of cfDNA samples from healthy individuals using GATK’s *HaplotypeCaller* (Poplin *et al.*, 2017). If a panel of healthy individuals is unavailable, the user may instead provide a panel composed of all available matched controls for this step. Genotyping results are then supplied to GATK’s *VariantFiltration* method for filtering out any call that has read support in more than a specified percentage of samples (default: 10% of the panel of normals) (Poplin *et al.*, 2017). All resulting calls are converted to table format using GATK’s *VariantsToTable* command with parameters that can be customized by the user (https://github.com/broadinstitute/gatk).

Aspects of PACT’s SNV/indel workflow are comparable to the McDonnell Genome Institute’s public analysis workflow which has also been distributed in CWL format (<https://github.com/genome/analysis-workflows>).

***CNA workflow***

For CNA analysis, a read depth-based method was first employed to calculate the log-transformed ratio of depth between the patient cfDNA sample and the control panel based on cfDNA samples from healthy individuals or matched control normal samples, corrected for biases in GC content and repeat content. If a panel of healthy individuals is unavailable, the user may instead provide a paenl composed of all available matched controls. We employed CNVKit for read depth ratio calculation, control panel construction and bias correction (Talevich *et al.*, 2016). Next, log ratio of depth was recentralized using the CN control regions (chosen as the least CN altered regions via surveying existing whole genome sequencing data, if available) to account for depth bias often seen in targeted sequencing of cfDNA. Finally, regions with log ratio of depth that deviated from that of the CN controls (default: 3 standard deviations from the mean) were called CNAs.

**Benchmarking**

***Application to published cfDNA prostate cancer cohort***

Blood plasma cfDNA sequencing data from published prostate cancer patients that had been reported to contain either 1) tandem duplications of the Androgen Receptor (or its enhancer) or 2) deletions resulting in TMPRSS2::ERG gene fusions were selected for initial PACT testing (Dang, Chauhan, *et al.*, 2020). Although matched tissue WGS was available for only one patient with a reported SV (WGS supported the SV found in cfDNA for this patient), calls were considered reliable as these two SVs are well recognized hallmarks in prostate cancer and, in this cohort, correlated with survival outcomes. Sensitivity was assessed based on the total number of previously reported SVs detected by each tool. False positive rates were not evaluated due to the lack of validation sequencing of novel calls. All tools were run with default settings, with the exception of SViCT, which was run using *‘-M 6000000’* (default: *-M 2000*). The -M parameter defines the max size of SVs evaluated by SViCT and was increased based on the reported size of the previously detected events.

***Application to Horizon Discovery reference data***

The Horizon Discovery reference dataset has been sequenced and is expected to contain *SLC34A2*::*ROS1* and *CCDC6*::*RET* gene fusions, both at approximately 5% allele frequency (Catalog number: HD786, SRA: SRR8551544). Fastq files were downloaded and aligned by *bwa mem* (Li, 2013) with default settings and duplicate reads were marked using the Picard *MarkDuplicate* tool(Broad Institute, 2019). Outputs were then sorted using sambamba 0.6.8 (Tarasov *et al.*, 2015). All tools were run on the sorted bam files using default settings. As no panel of healthy normals was available for use with PACT, we substituted it with a panel of matched controls. Unfortunately, SViCT repeatedly failed to run to completion, despite the standard bam format of the input files. Specifically, SViCT crashed with a “double free or corruption (!prev)” error message. With repeated testing with or without changes to the ‘-M’ parameter, we repeatedly received either this error or at times an exit code 139 error (segmentation fault). Both kinds of errors are standard C++ error messages (SViCT is written in C++) and usually means that there was an error within the software itself that caused the program to mismanage its memory resources. We noted that multiple issues have been opened on the project’s GitHub page relating to these and other memory related error messages. It is unclear why the tool ran successfully on the cfDNA prostate cancer cohort (suggesting the tool was installed correctly) but failed on these samples, even though these samples were compatible with all other tested tools. For these reasons, we excluded SViCT from this analysis. Performance of the remaining tools was assessed by checking for the detection of the reported *SLC34A2::ROS1* and *CCDC6::RET* fusions and by determining the number of reported SVs that were called but were not expected to appear in this particular Horizon Discovery dataset.

In addition to validated SVs, the Horizon Discovery dataset includes a number of validated SNVs, INDELS and CNAs. We evaluated whether PACT could detect these events as well, in addition to the SVs. However, the SNV/INDEL and CNA workflows within PACT are composed of methods already widely used in the ctDNA space and were included in PACT primarily for convenience, so comprehensive benchmarking was not performed. We found that PACT detected 100% of SNVs/INDELs in this data (Supplementary Table S3). In addition to the 5 validated events, PACT reported an additional 21 non-synonymous variants. Horizon Discovery provides variant calls performed on the genomic DNA version of this product (Catalog number: HD753) and we were able to confirm that all 21 of these variants are present in the genomic data (but had not been validated in their official ctDNA reference). No false positives were present.

***In silico simulation***

*In silico* simulation data were generated using sequencing data from solid tumor samples collected from two different cohorts. The first was the prostate cancer cohort described in Dang, Chauhan, *et al*., 2020. We selected 4 solid tumor prostate samples based on the criteria that *TMPRSS::ERG* fusions had been detected 1) in the solid tumor data and 2) in cfDNA from the same patient. Although validation sequencing was not performed, the presence of this well documented fusion in multiple samples from the same patients were considered sufficient for them to be treated as true positives. The second cohort was a colorectal cancer cohort described in Dang, Krasnick, *et al*., 2020. In that study, whole genome sequencing was followed by targeted validation sequencing on a number of solid tumor samples. We selected 5 samples, which contained a total of 7 validated SVs, based on the criteria that 1) the same sample was used for both discovery and validation sequencing and 2) the SV had >50 supporting reads in the validation sequencing. A summary of the selected samples and monitored SVs are described in Supplementary Table S2. All selected samples also had matched control data available.

Sequencing reads from all samples were aligned and processed as was done with the Horizon Discovery data. Reads from aligned tumor data were then systematically combined with reads from the aligned reads of each sample’s respective matched control. Calculations for the number of reads used when generating samples of different tumor DNA content levels were performed based on the total number of reads in a sample (based on *samtools flagstat* output) (Danecek *et al.*, 2021) and the previously annotated tumor purity of each tumor sample (Dang, Chauhan, *et al.*, 2020; Dang, Krasnick, *et al.*, 2020). Reads were downsampled from each tumor sample using Picard *DownsampleSam* and then downsampled reads from each tumor were merged with their respective matched control using Picard *MergeSamFiles* (Broad Institute, 2019). Combined files were then labeled internally based on their expected dilution using the Picard *AddOrReplaceReadGroups* command and output bam files were checked with *samtools flagstat* to confirm that they contained the expected number of reads (Broad Institute, 2019; Danecek *et al.*, 2021). This process was repeated 100 times for each sample at each tested tumor DNA content level, using a random seed each time for the *DownsampleSam* command.

All simulated samples were analyzed using the default settings of each tool and sensitivity was assessed by determining the number of previously selected (and validated) SVs detected at each tumor DNA content level across all 100 iterations. False positive rates were not assessed as we were unable to perform validation sequencing on potential novel calls. We were unable to assess SViCT’s performance as it failed to run to completion on any of the simulated samples, failing on each sample with the same errors observed when the tool was applied to the Horizon Discovery dataset.

***In vitro experiment***

We further evaluated PACT performance in an *in vitro* dilution experiment of a breast cancer cell line (HCC1395). First, published genomic breakpoints from 26 validated gene fusions in the HCC1395 breast cancer cell line were used to design a targeted panel using Roche’s HyperDesign tool (https://hyperdesign.com). HCC1395 cancer cells were then combined *in vitro* with the matched control cell line (HCC1395BL) following a standard serial dilution strategy to simulate different tumor DNA content levels (0.1%-100%) and then sequenced isolated DNA samples using our targeted panel. Sequencing reads were UMI tagged and aligned using *bwa mem*. Reads were grouped by UMI (*fgibio GroupReadsbyUmi*) and consensus reads were called (*fgibio CallDuplexConsensusReads*) (FulcrumGenomics, 2022) to reconstruct the most likely read representing the corresponding DNA fragment. Consensus reads were then re-aligned using *bwa mem* for final alignment *(Li, 2013)*.

All samples were analyzed with each SV caller using default settings. SVs targeted by potentially poorly designed probes were removed from analysis by filtering out any SV without any coverage at the 100% tumor DNA content level (8 SVs). Similarly, samples with <0.12% tumor DNA content were removed from analysis as no tool made a correct call at that level. Again, we were unable to get SViCT to run to completion on the HCC1395 samples, despite the files being compatible with all other tools and instead received the same error messages as previously described. As no panel of healthy normals was available, we used a panel of matched controls. To demonstrate the benefit of using control data, we also attempted to run PACT without using any controls (Supplementary Figure S6). Although PACT requires controls, were able to run PACT without controls by using the Rsubread R package to simulate sequencing reads that aligned to a chromosome not included in the original targeted panel. By doing so, we were able to provide minimalistic bam files of untargeted regions, rather than actual control samples. We confirmed that no SVs were filtered based on support found in these minimalist bam files and found that PACT outperformed other tools at most tumor DNA content levels even without the benefit of control data.

For evaluation, all calls that did not match previously validated SVs were labeled as false positives (FP) and validated calls that were not detected were labeled as false negatives (FN). Similarly, true positives were defined as calls that matched validated SVs. Sensitivity was used to assess the proportion of true events that were detected and precision was used to assess the proportion of all calls made that corresponded to true events. The F1 accuracy score is the harmonic mean of the sensitivity and precision, such that a result with perfect precision and sensitivity would have an F1 score of 1.0 and poor performance would have a score that approaches 0. Performance metrics were calculated using the formulas:

$Sensitivity= \frac{TP}{TP+FN}$ (1)

$Precision= \frac{TP}{TP+FP}$ (2)

$F1 Score=2* \frac{Precision*Sensitivity}{Precision+Sensitivity}$ (3)

***Resource Requirements***

PACT is designed to be used in a high-performance computing (HPC) environment. The pipeline itself contains a variety of published bioinformatics tools and therefore its minimum computing requirements are determined by the most resource intensive tool in the pipeline (CNVkit), which is set to require 64GB of RAM and 12 cores. Minimum requirements for any given tool can be manually changed by modifying the CWL file wrapper for the tool found in the *tools* directory on the project GitHub page, although we believe we have provided sensible default requirements for all tools. We found CPU time to be highly variable and it may be influenced by many factors including (but not limited to) sequencing depth, number of samples/matched controls, number of healthy normals, and number of variants identified. For reference, we found the median CPU time across the repeated iterations in our SV analysis of the *in silico* simulation to be 16.49 seconds, with an average of 192.14 seconds. In that analysis, samples had a median of 5.9 million reads (average of 7.4 million).

**References**

Ainscough,B.J. *et al.* (2016) DoCM: a database of curated mutations in cancer. *Nat Methods*, **13**, 806–807.

Broad Institute (2019) Picard Toolkit.

Chen,X. *et al.* (2016) Manta: rapid detection of structural variants and indels for germline and cancer sequencing applications. *Bioinformatics*, **32**, 1220–1222.

Chiang,C. *et al.* (2015) SpeedSeq: ultra-fast personal genome analysis and interpretation. *Nat Methods*, **12**, 966–968.

Cibulskis,K. *et al.* (2013) Sensitive detection of somatic point mutations in impure and heterogeneous cancer samples. *Nat Biotechnol*, **31**, 213–219.

Cingolani,P. *et al.* (2012) A program for annotating and predicting the effects of single nucleotide polymorphisms, SnpEff: SNPs in the genome of Drosophila melanogaster strain w1118; iso-2; iso-3. *Fly (Austin)*, **6**, 80–92.

Danecek,P. *et al.* (2021) Twelve years of SAMtools and BCFtools. *GigaScience*, **10**, giab008.

Dang,H.X., Chauhan,P.S., *et al.* (2020) Cell-Free DNA Alterations in the AR Enhancer and Locus Predict Resistance to AR-Directed Therapy in Patients With Metastatic Prostate Cancer. *JCO Precision Oncology*, 680–713.

Dang,H.X., Krasnick,B.A., *et al.* (2020) The clonal evolution of metastatic colorectal cancer. *Sci Adv*, **6**, eaay9691.

FulcrumGenomics (2022) fgibio.

Jeffares,D.C. *et al.* (2017) Transient structural variations have strong effects on quantitative traits and reproductive isolation in fission yeast. *Nat Commun*, **8**, 14061.

Khanna,A. *et al.* (2022) Bam-readcount - rapid generation of basepair-resolution sequence metrics. *JOSS*, **7**, 3722.

Kim,S. *et al.* (2018) Strelka2: fast and accurate calling of germline and somatic variants. *Nat Methods*, **15**, 591–594.

Koboldt,D.C. *et al.* (2009) VarScan: variant detection in massively parallel sequencing of individual and pooled samples. *Bioinformatics*, **25**, 2283–2285.

Larson,D. *et al.* (2019) hall-lab/svtools: svtools v0.5.1.

Layer,R.M. *et al.* (2014) LUMPY: a probabilistic framework for structural variant discovery. *Genome Biol*, **15**, R84.

Li,H. (2013) Aligning sequence reads, clone sequences and assembly contigs with BWA-MEM.

McLaren,W. *et al.* (2016) The Ensembl Variant Effect Predictor. *Genome Biol*, **17**, 122.

Poplin,R. *et al.* (2017) Scaling accurate genetic variant discovery to tens of thousands of samples Genomics.

Rausch,T. *et al.* (2012) DELLY: structural variant discovery by integrated paired-end and split-read analysis. *Bioinformatics*, **28**, i333–i339.

Talevich,E. *et al.* (2016) CNVkit: Genome-Wide Copy Number Detection and Visualization from Targeted DNA Sequencing. *PLoS Comput Biol*, **12**, e1004873.

Tan,A. *et al.* (2015) Unified representation of genetic variants. *Bioinformatics*, **31**, 2202–2204.

Tarasov,A. *et al.* (2015) Sambamba: fast processing of NGS alignment formats. *Bioinformatics*, **31**, 2032–2034.

Ye,K. *et al.* (2009) Pindel: a pattern growth approach to detect break points of large deletions and medium sized insertions from paired-end short reads. *Bioinformatics*, **25**, 2865–2871.


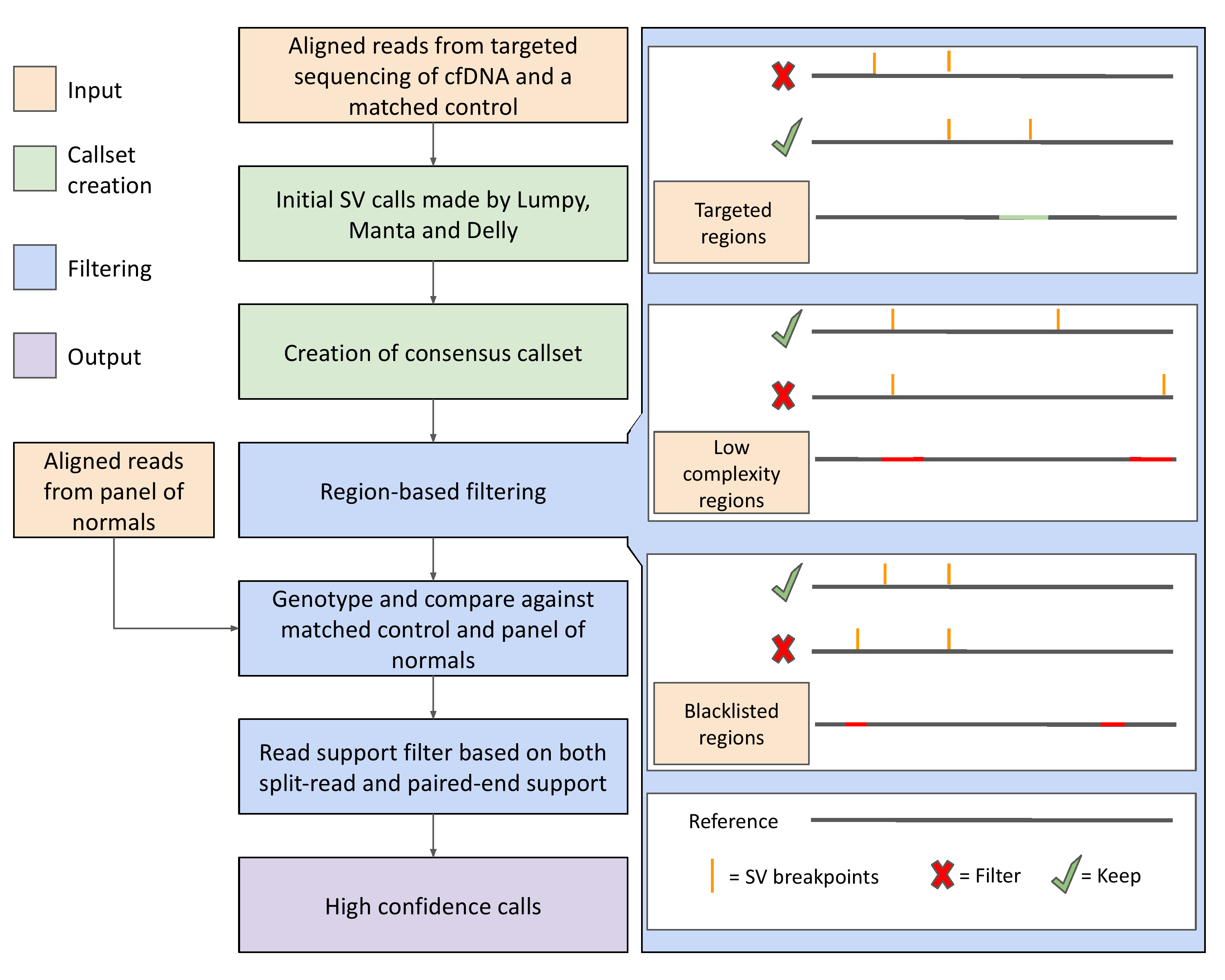


**Supplementary Figure S1.** Overview of SV-calling portion of PACT. Aligned reads from targeted sequencing of cfDNA and a matched control are analyzed by an ensemble of SV callers using sensitive settings and consensus calls are then identified. A variety of filtering steps are then applied to reduce expected cfDNA noise. Region-based filters require that at least one breakpoint corresponds to a region targeted by the sequencing panel and filters out potential sequencing errors by removing SVs with more than one breakpoint that corresponds to low complexity genomic regions and SVs with any breakpoints that originate in blacklisted regions. Remaining candidates are then genotyped in a panel of healthy unmatched individuals to further remove potential artifacts and germline events, and then read support filtering is finally applied.

**
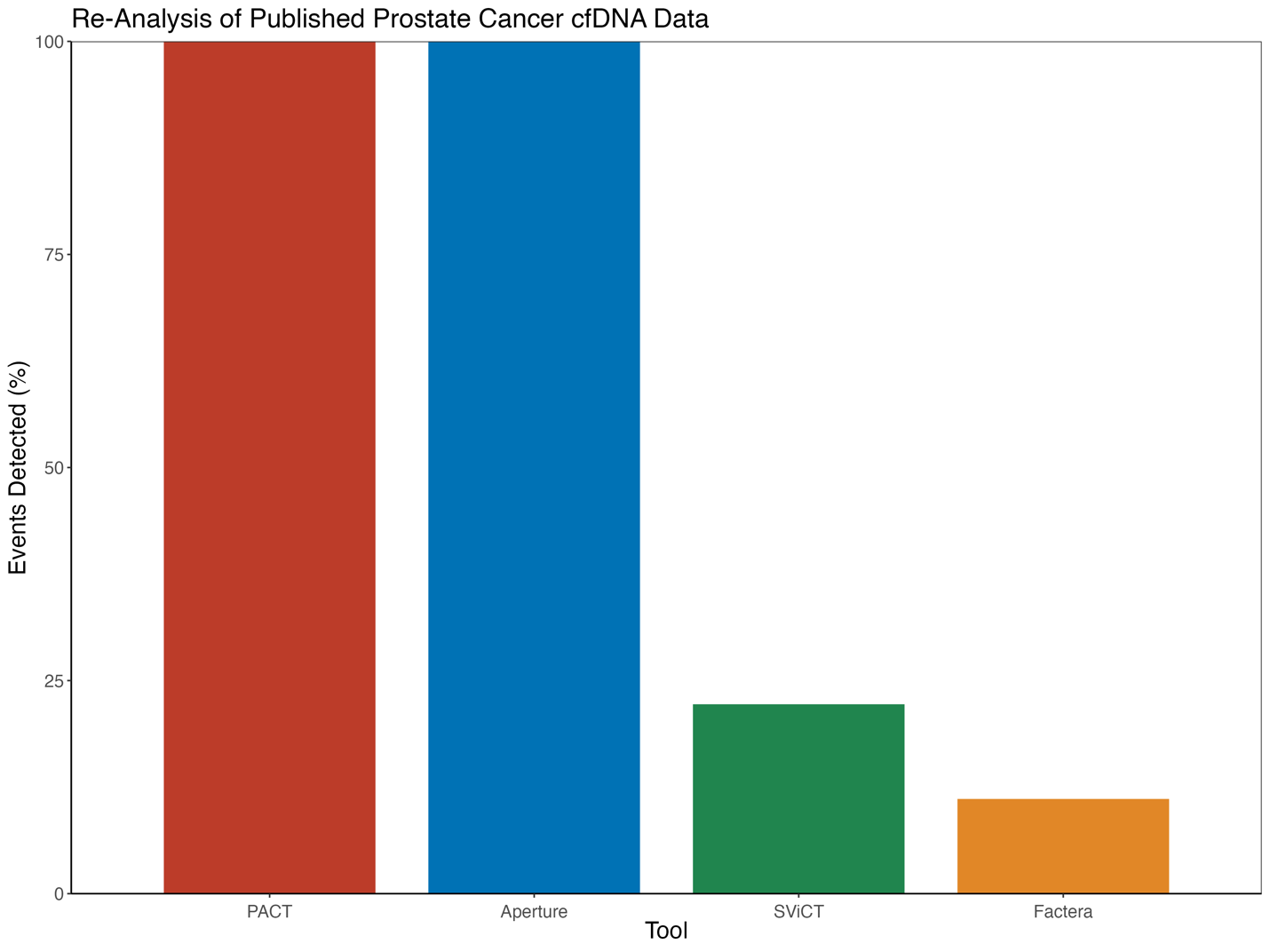
**

**Supplementary Figure S2.** Re-analysis of published cfDNA data from an advanced prostate cancer cohort. Events detected are based on 5 tandem duplications of the Androgen Receptor and/or its upstream enhancer and 4 deletions resulting in TMPRSS2::ERG gene fusions. Both events are considered hallmarks of prostate cancer and correlated with survival in the original publication.


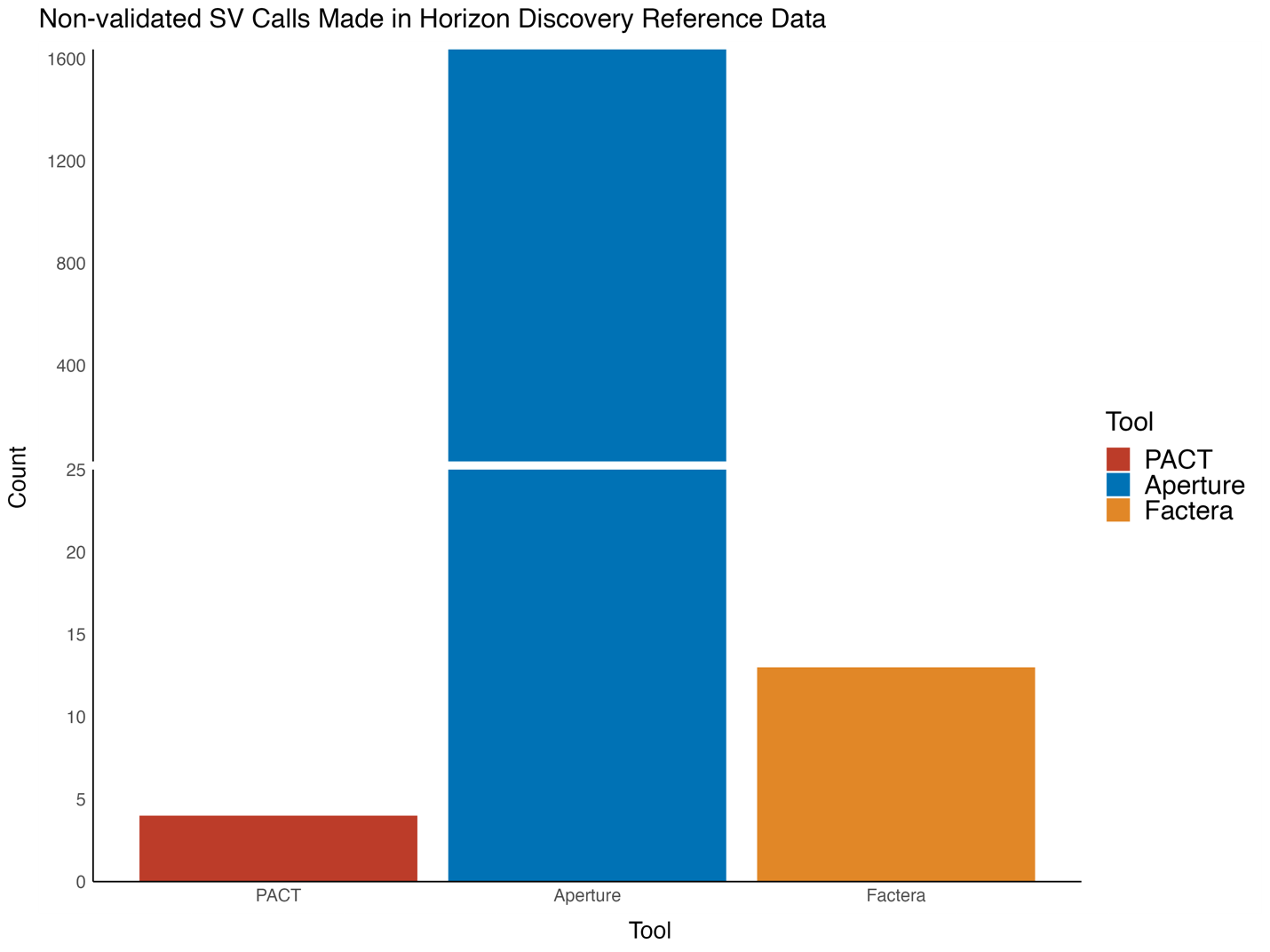


**Supplementary Figure S3.** Number of SVs reported that do not match previously validated SVs as reported by Horizon Discovery in their cfDNA reference dataset (SRA: SRR8551545). SViCT results are not shown as it failed to run to completion.


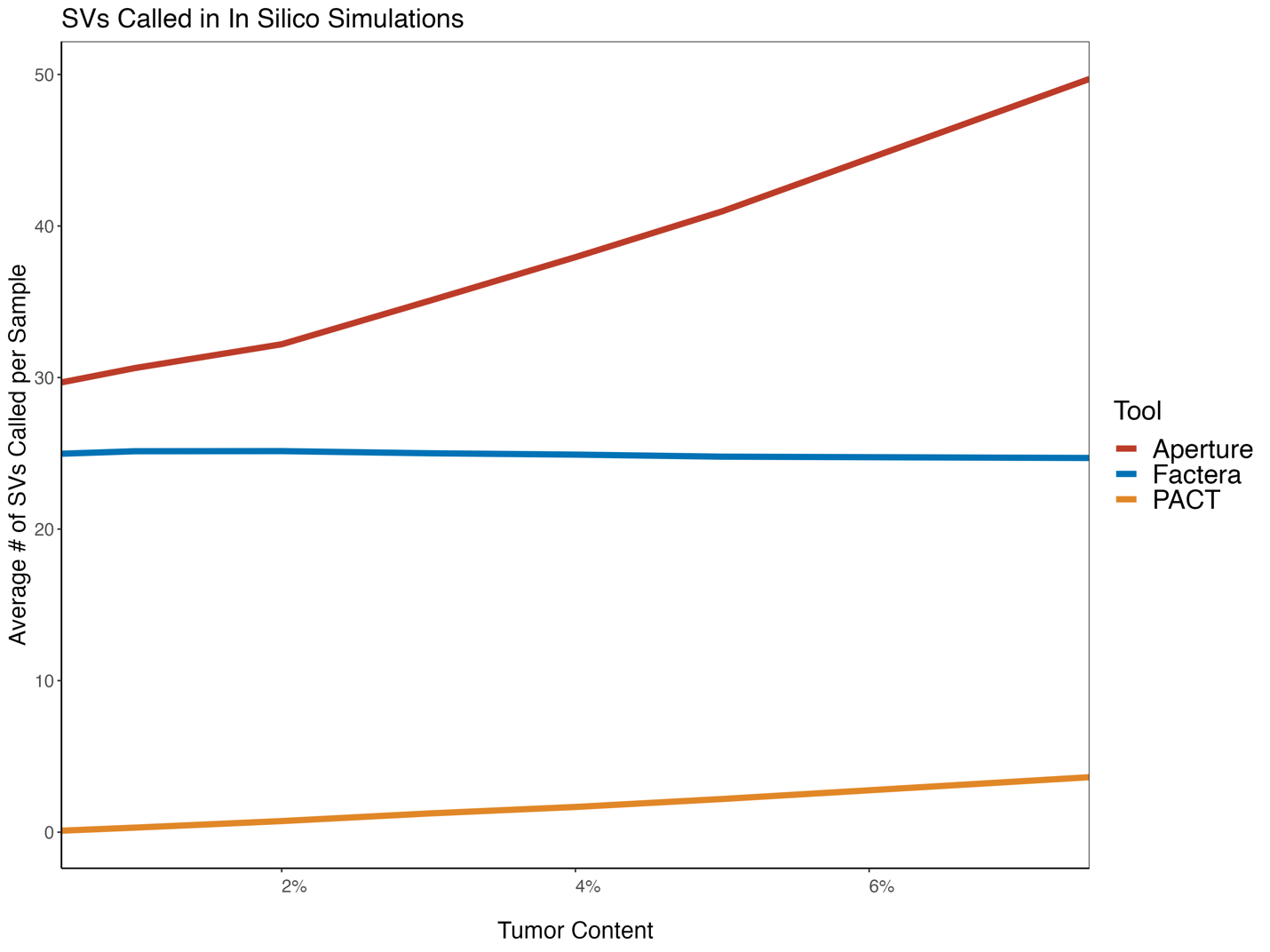


**Supplementary Figure S4**. Average number of SVs called per sample during *in silico* simulations based on prostate and colorectal cancer samples. Averages are based on 9 samples used across 100 iterations of the simulation. Only dilutions of <=7.5% tumor content are shown, as some samples were unable to be simulated at higher content levels due to the low tumor purity of the original samples being used as the basis for the simulation. Precision was not formally calculated due to the lack of validation sequencing of novel calls. However, PACT simultaneously achieved the highest sensitivity in these samples (Fig. 1B) and also reported the fewest total SVs, suggesting high precision.


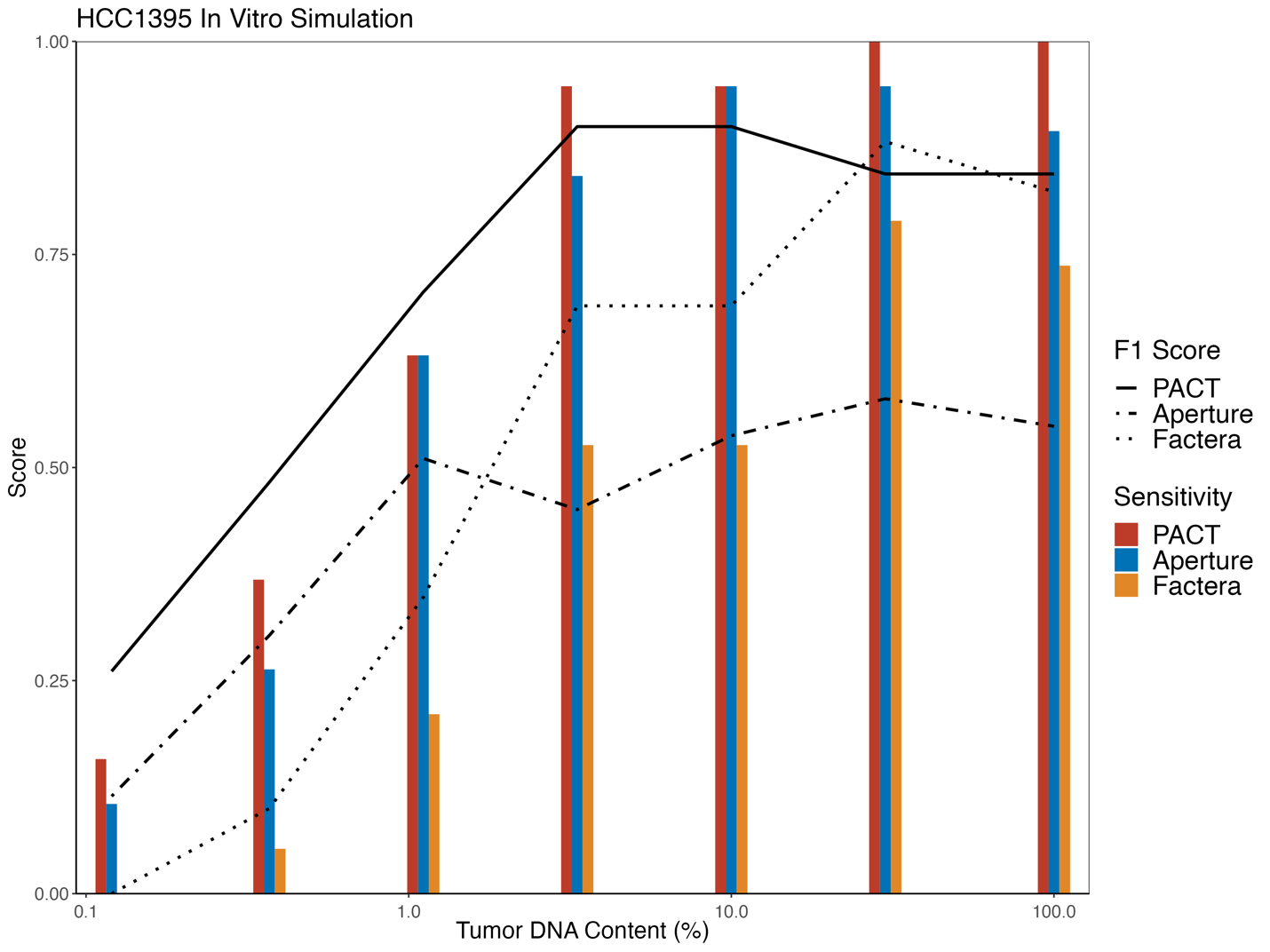


**Supplementary Figure S5.**  Sensitivity and F1 accuracy scores across different tumor DNA content levels based on in vitro simulation using the HCC1395 breast cancer cell line. SViCT data is not shown as the tool failed to run to completion.


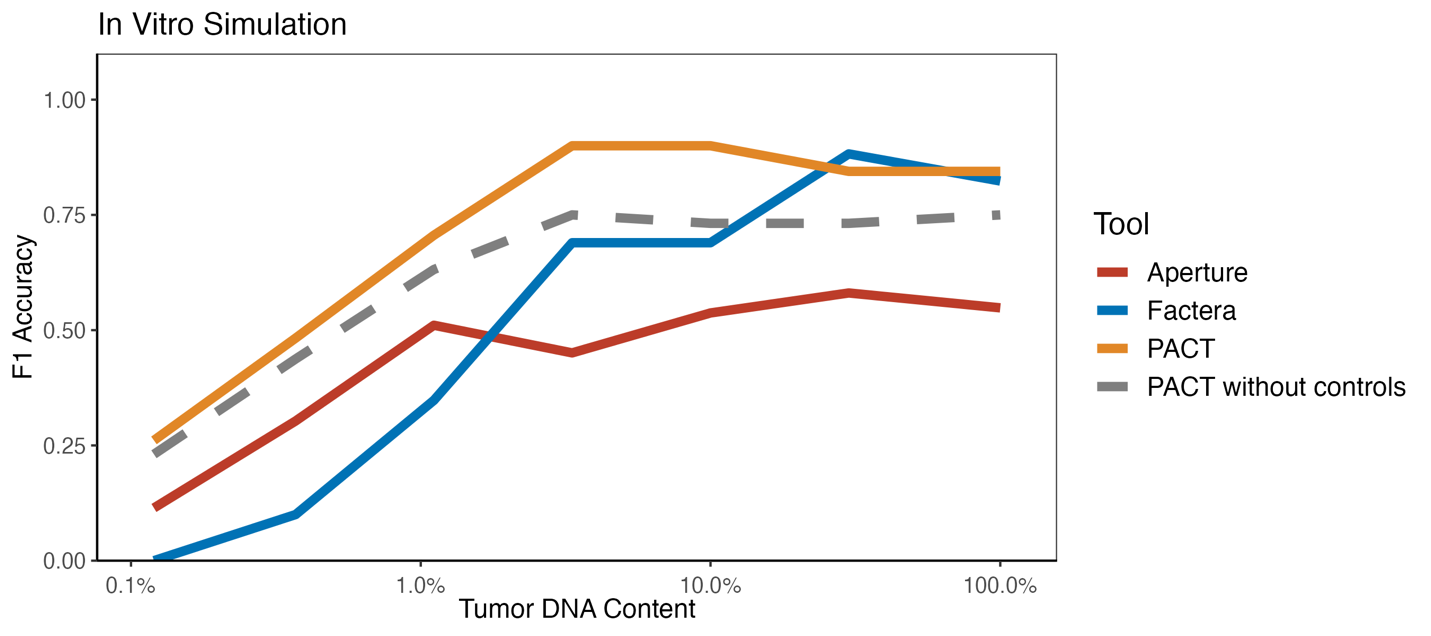


**Supplementary Figure S6.** Analysis of in vitro data without the use of control data demonstrates the benefit of including control data samples. Results suggest that although PACT benefits from the use of control data, it still has performance comparable to Aperture and Factera when proper control data is unavailable.

| **Tool** | **SV Types Called** | **SV Size Limitations** | **Accepts Matched Control** | **Publication** |
| --- | --- | --- | --- | --- |
| SViCT | All | <2kb | No | Gawronski, et al. *Nuc Acid Res*. 2019 |
| Factera | Gene fusions | None | No | Newman, et al. *Bioinformatics: App Notes.* 2014 |
| Aperture | Does not label SVs by type | None | No | Liu, et al. *Briefings in Bioinformatics*. 2021. |

**Supplementary Table S1.** Descriptions and limitations of published ctDNA SV callers

| **Cancer of Origin** | **Structural Variant** | **# of Samples** |
| --- | --- | --- |
| Prostate | *TMPRSS2*::*ERG* | 4 |
| Colorectal | *VIT1A*::*TCF7L2* | 2 |
| Colorectal | *STRAP*::*DERA* | 1 |
| Colorectal | *PDE4D*::*SEC24A* | 1 |
| Colorectal | *IFT11*::*RHO* | 1 |
| Colorectal | *BIRC6*::*PLB1* | 1 |
| Colorectal | *ABR*::*NAALADL2* | 1 |

**Supplementary Table S2.** Description of samples used for *in silico* simulation and the previously validated structural variants contained in those samples. All selected structural variants result in gene fusions. Total of 4 prostate and 5 colorectal samples, with 2 of the colorectal samples having 2 validated fusions.

| **Gene** | **Mutation** | **Detected by PACT** |
| --- | --- | --- |
| GNA11 | c.626A>T | Yes |
| AKT1 | c.49G>A | Yes |
| PIK3CA | c.1633G>A | Yes |
| EGFR | c.2300_2308dup | Yes |
| EGFR | c.2235_2249del | Yes |
| MYC | Amplification | Yes |
| MET | Amplification | Yes |

**Supplementary Table S3.** All validated SNVs, INDELS and CNVs, as reported by Horizon Discovery, in the reference cfDNA dataset and their detection status based on the SNV/CNV workflows found in PACT. PACT reported an additional 21 non-synonymous variants in this dataset, all of which have been reported by Horizon Discovery in the genomic DNA that is meant to correspond to their ctDNA reference, suggesting that PACT was able to detect additional true SNVs that had been validated in the genomic DNA, but not ctDNA, version of this reference.
